# Investigation of the Phageome and Prophages in French Cider, a Fermented Beverage

**DOI:** 10.3390/microorganisms10061203

**Published:** 2022-06-12

**Authors:** Pierre Ledormand, Nathalie Desmasures, Cédric Midoux, Olivier Rué, Marion Dalmasso

**Affiliations:** 1 Normandie Univ., UNICAEN, UNIROUEN, ABTE, 14000 Caen, France; pierre.ledormand@unicaen.fr (P.L.); nathalie.desmasures@unicaen.fr (N.D.); 2 INRAE, MaIAGE, Université Paris-Saclay, 78350 Jouy-en-Josas, France; cedric.midoux@inrae.fr (C.M.); olivier.rue@inrae.fr (O.R.); 3 INRAE, BioinfOmics, MIGALE Bioinformatics Facility, Université Paris-Saclay, 78350 Jouy-en-Josas, France; 4 INRAE, PROSE, Université Paris-Saclay, 92761 Antony, France

**Keywords:** phages, phageome, prophage, metagenomics, fermented beverages, cider

## Abstract

Phageomes are known to play a key role in the functioning of their associated microbial communities. The phageomes of fermented foods have not been studied thoroughly in fermented foods yet, and even less in fermented beverages. Two approaches were employed to investigate the presence of phages in cider, a fermented beverage made from apple, during a fermentation process of two cider tanks, one from an industrial producer and one from a hand-crafted producer. The phageome (free lytic phages) was explored in cider samples with several methodological developments for total phage DNA extraction, along with single phage isolation. Concentration methods, such as tangential flow filtration, flocculation and classical phage concentration methods, were employed and tested to extract free phage particles from cider. This part of the work revealed a very low occurrence of free lytic phage particles in cider. In parallel, a prophage investigation during the fermentation process was also performed using a metagenomic approach on the total bacterial genomic DNA. Prophages in bacterial metagenomes in the two cider tanks seemed also to occur in low abundance, as a total of 1174 putative prophages were identified in the two tanks overtime, and only two complete prophages were revealed. Prophage occurrence was greater at the industrial producer than at the hand-crafted producer, and different dynamics of prophage trends were also observed during fermentation. This is the first report dealing with the investigation of the phageome and of prophages throughout a fermentation process of a fermented beverage.

## 1. Introduction

Bacteriophages (phages) are viruses infecting bacteria, and that can establish lytic, chronic or temperate (lysogenic) infections. To designate phage communities, the term “phageome” is frequently used in the scientific literature. However, it seems that no consensual definition of this term has been retained to date. In some cases, the term “phageome” seems to encompass communities of free lytic phages and of prophages (temperate phages included in bacterial genomes) [1]. Nevertheless, the majority of the studies dealing with phageomes often consider free lytic phages, without including prophages [2,3]. Consequently, the term “phageome” will be used in the current study for designating free lytic phage communities only. Prophages will be considered separately. Phageomes are studied in multiple environments such as the human gut [1,2,3], the human oral cavity [4], plants [5], oceans [6], and more recently, glaciers [7]. Studies of the phageomes in fermented foods and beverages are scarce [8]. To date, only three studies strictly describing the phageomes of Asian fermented food products such as kimchi, sauerkrauts and fermented shrimps [9,10], and of a French cheese [11] are available, but none about fermented beverages. Environmental sources of phages in cheese have also been studied by investigating the airborne viromes (whole virus communities) in two cheese production plants [12]. Prophages (temperate phages) are also well known for their impact on the functioning of the bacterial cells that carry them, either through beneficial or non-beneficial interactions [13]. Their impact at the scale of a microbial community remains underexplored, and still needs to be taken into account [14]. In this way, exploring the diversity of prophages also seems essential to assess their role in microbial fluxes within ecosystems such as fermented foods and beverages, as phages are recognised to be the drivers of microbial diversity [15].

Although the phageomes of fermented foods are not well described yet, their presence in such matrices has been known for decades. Lytic phages are extensively studied for their antibacterial properties against pathogenic bacteria encountered in the food industry such as, for example, *Salmonella* sp. [16] and *Listeria monocytogenes* [17], and against spoilage bacteria such as *Acetobacter* in wine [18] and *Levilactobacillus brevis* in beer [19]. Lytic phages are also known as killers of such industrial starters as in dairy products, where phages targeting *Lactococcus lactis* and *Streptococcus thermophilus* [20] can cause fermentation defects. No study is available about the phageomes in fermented beverages, especially in cider. Cider is an alcoholic fermented apple beverage, consumed all around the world, presenting harsh physico-chemical conditions with low pH values (3.0–4.0) and the presence of ethanol [21], polyphenols and volatiles [22]. Two types of fermentation occur during cider elaboration. First, the alcoholic fermentation is performed by yeasts, including the *Saccharomyces* species (*S. cerevisiae* and *S. uvarum*) and non-*Saccharomyces* species (*Hanseniaspora*, *Candida*) [21]. Then, the malolactic fermentation (MLF) is performed by Lactic Acid Bacteria (LAB) belonging to *Oenococcus* sp., *Leuconostoc* sp., *Pediococcus* sp. and *Lactobacillus sensu lato* [22]. Describing phageome diversity in cider is thus interesting to better understand the microbial dynamics during the fermentation processes. The aim of this work was to study the diversity of the phageome, and also the prophage distribution in cider, throughout the fermentation process in two cider tanks from two producers. Several methods were thus tested to extract free phage particles from cider, in order to explore the phageome, and prophages were searched for inside bacterial metagenomes throughout the cider fermentation.

## 2. Materials and Methods

### 2.1. Cider Sample Collection for Phageome and Prophage Investigation

A tank from an industrial producer (B) and a tank from a hand-crafted producer (P), located in the Normandy region (France), were sampled during the first month of the cider fermentation process, at four time points, to explore the presence of free phage particles and of prophages. Details about the collected samples are given in Table 1.

### 2.2. Methodological Developments for Collecting Phage Particles from Cider and for Phageome DNA Extraction

To collect and extract total phage DNA from cider, different approaches were tested and are summarised in Table 2.

#### 2.2.1. Validation of Methods on Cider Samples Spiked with Phages

First, phageome DNA extraction was tested on cider samples spiked with phage UCMA 21115, previously isolated from cider [23] and originating from the UCMA collection (Université de Caen Microbiologie Alimentaire, Caen, France), in order to validate the method used. Concentrations of phage UCMA 21115 ranging from 10^4^ to 10^9^ PFU/mL were used (Table 2—**I**, **II** and **III**). Spiked cider samples (50 mL) were centrifuged at 4700× *g* for 20 min at 4 °C, and the pellet was suspended into 500 µL SM buffer (200 mM NaCl, 10 mM MgSO_4_, 50 mM Tris pH 7.5) (Table 2—**I**). Phage precipitation with PEG-8000 (*w*/*v*) and 0.5 M NaCl was also tested by adding them directly to spiked cider, adjusted (Table 2—**III**) or not (Table 2—**II**) to pH 5.5. PEG-8000 addition was followed by an incubation step at 4 °C overnight to maximise the precipitation of phage particles. Then, samples were centrifuged, as described above, and the pellets were suspended into 500 µL SM buffer.

In all cases, one volume of chloroform was added to the pellets or to the precipitates suspended into 500 µL SM buffer beforehand, in order to eliminate membrane vesicles [11]. Samples were then centrifuged at 8000× *g* for 5 min. The aqueous phase was treated with DNAse (Fisher Bioreagents, Vilnius, Lithuania) and RNAse (Thermofisher, Vilnius, Lithuania) for 1 h at 37 °C to eliminate non-viral DNA. Then, 10% SDS and 20 mg/mL proteinase K (Thermofisher, Vilnius, Lithuania) were added and an incubation step at 56 °C for 20 min was performed [24]. Finally, phage DNA were extracted with three different methods. The first one (named buffer 1), based on a method described for faecal samples, consisted in the addition of 100 µL Phage Lysis Buffer (4.5 M guanidinium isothiocyanate, 44 mM sodium citrate pH 7.0, 0.88% sarkosyl, 0.72% 2-mercaptoethanol) [2]. In the two other methods, Phage Lysis Buffer was supplemented with 1% (buffer 2) or 2% (buffer 3) of polyvinylpolypyrrolidone (PVPP), in order to counteract the effects of cider polyphenols. These approaches were based on previous protocols designed for plant DNA extraction [25]. Samples were then incubated at 65 °C for 10 min.

Phage DNA purification was performed by two successive steps of phenol:chloroform:isoamyl 25:24:1 (Sigma Aldrich, St. Louis, MO, USA) (*v*/*v*) treatment and centrifugation at 8000× *g* for 5 min. DNA precipitation was performed with 2.5 volumes of absolute ice-cold ethanol before a centrifugation step at 13,000× *g* for 15 min at 4 °C. DNA was eluted in 50 µL of water, and quantified using a Nano TECAN spectrophotometer (Life Science, Zürich, Switzerland). DNA integrity was checked on a 1% agarose gel.

#### 2.2.2. Application to Non-Spiked Cider Samples

Phageome DNA extraction protocols were then applied to cider samples (not spiked with exogenous phage UCMA 21115), using different volumes (20, 50 and 1000 mL) (Table 2—**IV**, **V**, **VI**, **VII**). Cider samples were centrifuged at 4700× *g* for 20 min at 4 °C, and DNA extractions were performed on the pellets suspended into 500 µL SM buffer beforehand (Table 2—**IV**) and on the supernatants (Table 2—**V**) to maximise the chances of obtaining phage DNA. Supernatants were first filtered through 0.45 µm filters and precipitated overnight with PEG-8000 at 4 °C, before being centrifuged at 4700× *g* for 20 min at 4 °C. The resulting pellets were suspended into 500 µL SM buffer prior to DNA extraction, as described above.

DNA extractions were also directly performed after the addition of PEG-8000 and NaCl, prior to the first centrifugation step, on cider samples adjusted to pH 5.5 (Table 2—**VII**) or not (Table 2—**VI**). The precipitates were centrifuged as described above, and the pellets were suspended into 500 µL SM buffer. DNA was extracted following the same methods as described above.

#### 2.2.3. Optimisation of the Method to Collect Phage Particles from Cider

To concentrate phage particles from cider, 400 mL of cider was filtered with a tangential flow filtration (TFF) method (Table 2—**VIII**). The TFF system used was the Vivaflow^®^ 50 (Sartorius, Göttingen, Germany), a PES membrane cassette with a total surface area of 50 cm^2^ and a molecular weight cut-off of 100 kDa. In order to validate the functioning of the TFF system, cider was spiked with exogenous phage UCMA 21115, at a concentration of 10^5^ PFU/mL. Retentate was filtered through 0.45 µm filters before DNA extraction, as described above.

The second method used to concentrate phage particles was the chemical Fe-based flocculation, as described for the concentration of ocean viruses from large volume samples (Table 2—**IX**) [26]. As for TFF, 400 mL of cider spiked with exogenous phage UCMA 21115, at concentrations ranging from 10^4^ to 10^8^ PFU/mL, was tested. Briefly, FeCl_3_ was added to cider samples to obtain a final concentration of 1 mg/L before incubation at room temperature for 1 h to allow Fe-phage complexes to flocculate. Then, the solutions were filtered on Whatman polycarbonate membrane filters with 0.8 µm-pores. Finally, filter retentates were suspended into 10 mL suspension buffer (0.25 M ascorbic acid, 0.2 M Mg_2_EDTA, pH 6.0) before DNA extraction, as described above.

In parallel, phage isolation was also performed on these retentates from TFF and flocculation, potentially enriched in phages. Isolation and propagation were carried out on 160 strains from genera found in cider (Table S1), using the classical double layer plate technique with MRS 0.5% (*w*/*v*) supplemented with 10 mM CaCl_2_ and 10 mM MgSO_4_, and incubated at 30 °C for 24 h to allow for the formation of plaques.

### 2.3. Microbial DNA Extraction for Prophage Investigation

At each sampling time (Table 1), 50 mL of cider samples were centrifuged at 4700× *g* for 20 min at 4 °C. Total DNA from pellets was extracted and purified using the NucleoSpin Soil kit (Macherey Nagel, France) according to the manufacturer’s instructions. DNA quality and quantity were checked as described above.

### 2.4. DNA Sequencing and Bioinformatics Analysis

DNA library preparation and sequencing were carried out at Genewiz facilities (Leipzig, Germany), on an Illumina HiSeq sequencer producing paired-end reads of 2 × 150 bp in length. To access the quality of raw reads FastQC v.0.11.9 [27] was used. FastP v 0.20.0 [28] was used to eliminate reads with a length inferior to 50 nucleotides and bases with a bad quality (lower than 15). Sequence assembly was performed with SPADES v 3.14.0 with option—meta [29]—and only contigs longer than 1 kb were kept. Moreover, taxonomic assignations to access the composition of trimmed reads was done using kaiju v 1.7.3 [30] against database nr_euk (103 M protein sequences from nr: Bacteria, Archaea, Viruses, Fungi and microbial eukaryotes). The sequencing data from this study are available in NCBI SRA repository, reference number PRJNA803977.

### 2.5. Prophage Prediction

VIBRANT was used for prophage prediction [31]. VIBRANT is a hybrid machine learning used to maximise identification of lytic viral genomes and integrated proviruses, including highly diverse viruses. VIBRANT was used separately for each sample and contigs identified with VIBRANT were clustered between all samples using cd-hit-est [32] with default parameters (global sequence identity 90%). Reads were finally mapped on this set of contigs identified as potential phages. To generate abundance matrices, mapping results were converted into reads per length of the contig (in kb) per million (RPKM) and RPKM values of contigs above 5.0 were kept for analyses. Contigs identified by VIBRANT were aligned with BLAST against the NCBI non-redundant nucleotide database (E-value < 10^−16^ and percentage of identity >90%) to generate information about the host.

## 3. Results

### 3.1. Development of Methods for Phageome DNA Extraction from Cider

In order to explore the diversity of the phageome of cider, the total phage DNA was intended to be extracted from cider samples. Consequently, several methods for the collection of phages were tested (Table 2). For each type of sample preparation, three DNA extraction methods were performed (buffers 1, 2 and 3).

#### 3.1.1. Validation of the Extraction Method after Collection of Phage Particles from Spiked Cider Samples

Phageome DNA extraction methods adapted from other matrices [2,11] were first tested on cider samples spiked with phage UCMA 21115 [23], at concentrations ranging from 10^4^ to 10^9^ PFU/mL. In some cases, the pH of the samples was adjusted to 5.5 (Table 2—**III**–**VII**), as it corresponded to a pH value suitable for phage DNA extraction from cider in preliminary experiments (data not shown).

This step was also intended to determine the threshold of phages necessary to collect enough DNA in this matrix. In all the tested conditions, phage DNA was only extracted in cider samples adjusted to pH 5.5 after PEG precipitation (Table 2—**III**). At phage concentrations inferior to 10^7^ PFU/mL, no phage DNA was detected on agarose gel (Figure S1) whatever the DNA extraction buffer used, and very low quantities of DNA were obtained (less than 5–10 ng/µL). Phage DNA was detected at phage concentrations starting from 10^7^ PFU/mL whatever the DNA extraction buffer used (Figure S1). No phage DNA was extracted (data not shown) from spiked cider samples when the pH was not adjusted to 5.5 (Table 2—**I**,**II**). This result showed that phage DNA extraction methods were only efficient at a phage concentration starting from 10^7^ PFU/mL and at pH 5.5.

#### 3.1.2. Extraction of Phage Particles from Non-Spiked Cider Samples

The methods to collect phages and to extract the phageome DNA were then tested in non-spiked cider samples (Table 2—**IV**–**VII**). No phage DNA was extracted regardless of the tested buffers, the sample volume or the sample preparation (pellets, supernatants, cider concentrated with PEG and cider adjusted to pH 5.5 before PEG precipitation) (data not shown). As no phage DNA could be recovered in the non-spiked cider samples, whatever the tested conditions, it suggested a low occurrence of lytic phages in cider (probably less than 10^7^ PFU/mL), resulting in the difficulty to extract phageome DNA in such a fermented beverage.

#### 3.1.3. Viral Concentration Methods Were Tested to Improve the Phageome DNA Extraction

The previous results revealed a presumably low quantity of lytic phages in cider shown by the difficulty to extract phageome DNA from non-spiked cider samples. Thus, concentration methods were used in order to improve the phageome DNA extraction, starting from larger volumes of cider than the volumes tested above.

TFF was intended to be used to concentrate phages from up to 5 L of cider. A primary attempt was performed with a reduced batch of 400 mL of cider spiked with phage UCMA 21115 at a concentration of about 1.9 × 10^5^ PFU/mL (i.e., resulting in a total quantity of 7.6 × 10^7^ phages in 400 mL) as a positive control and to set the filtration parameters. At first, TFF appeared to be a suitable method to concentrate phages in cider samples because the total quantity of spiked phages was recovered in the retentate, with 8 × 10^7^ collected phages compared to the calculated 7.6 × 10^7^ expected phages. Concerning the filtration parameters, the flow rate was of 120 mL per hour for this cider sample, whereas it is rather around 750 mL per hour for sea water samples (supplier data). The attempts to filter (0.45 µm) the retentate (15 mL) to remove yeasts, bacteria and other cider components failed, as the cider retentate was too thick. As a result, phages were lost at this step with only 3.4 × 10^4^ phages that were recovered, which represents a loss of 3 log_10_. In view of these results, the option to use TFF in larger volumes of non-spiked cider samples was abandoned.

Another method to concentrate phages was tested, the iron (Fe)-based flocculation, which has the advantage of using large-pore-size filters (0.8 µm) compared to TFF. Concerning cider samples spiked with phage UCMA 21115, phage recovery was quite good at high phage concentrations. It was of 8.5 × 10^8^ for cider samples, for an initial inoculation of 4.0 × 10^10^, representing a phage loss of about ≈1 log_10_ (Table 3). At low phage concentrations, the phage recovery with Fe-based flocculation was less efficient. For example, with an initial inoculation of 4.0 × 10^6^, 5.0 × 10^3^ phage particles were retrieved in cider samples (Table 3). In addition, filtration of the cider sample was also quite complicated due to clogging. In view of these results, the option to use (Fe)-based flocculation on larger volumes of non-spiked cider samples was also abandoned.

In addition, the isolation of phages was attempted by using 160 potential host strains from genera found in cider, from the TFF retentate and after cider flocculation, to check the presence of potential lytic phages on these bacteria. No phage, except phage UCMA 21115 used as positive control, was retrieved from these samples, confirming that the phageome of cider was not abundant, and thus not easily accessible.

### 3.2. Prophage Investigation in Two Cider Tanks during Cider Fermentation

To complete the data concerning the lytic phageome, prophages present inside the metagenomes of the bacterial community involved in the cider fermentation process were investigated. As described in Table 1, cider sampling was carried out during the first month of cider fermentation in two tanks from two producers (B: industrial and P: hand-crafted). The prophage investigation was performed after a shotgun sequencing of whole genomic DNA, at each sampling time, through the exploration of prophage sequences in the bacterial metagenomic data. The number of reads was homogenous for all samples, with an amount of 2 × 10^7^ reads per sample, except for C1B3 which had more reads than the others (8 × 10^7^). The microbial composition of tanks B and P was of course accessible through these data, with tank P containing more reads assigned to Eukaryota than tank B (Figures S2 and S3, Table S2). Tank B presented a higher abundance in bacteria than tank P (Figure S2, Table S2). Within the bacteria, both tanks were dominated by the *Proteobacteria* phylum during the first three sampling times, while LAB appeared on the last sampling time (C1B4 and C2P4) (Figure S4).

After the phage prediction in the bacterial sequences with VIBRANT, 1603 contigs of putative phages were identified (114 were initially predicted as temperate and 1489 as lytic), the vast majority with a low- or medium-quality level. Among these contigs, only two complete prophages were identified, NODE_55 and NODE_129. The prophage NODE_55 was of 46,931 bases in length and matched with a *Podoviridae* CT3IJ1 (identity of 93%, coverage <1%, accession number BK023809.1) after a BLAST search against the NCBI virus database. The prophage NODE_129 was of 37,572 bases in length and matched with a *Siphoviridae* CTEVG3 (identity of 81%, coverage 16%, accession number BK027610.1) after a BLAST against the NCBI virus database. The 1603 phage contigs identified with VIBRANT were subjected to BLAST against the NCBI non-redundant nucleotide database (E-value < 10^−16^ and percentage of identity >90%) to generate information about the potential host. A total of 429 contigs (417 initially identified as lytic and 12 as temperate) gave no host identification according to the selected criteria, and were eliminated from the analysis of the prophages. So, 1174 contigs were retained, among them, 101 classified as temperate and 1073 as lytic by VIBRANT. Further analysis finally showed that all phage contigs matched with bacterial genomes and, therefore, were confirmed prophages. These results showed that the analysis of the life cycle by VIBRANT was a bit more uncertain in this case, probably due to the low-quality level of identification obtained.

**Figure 1:**
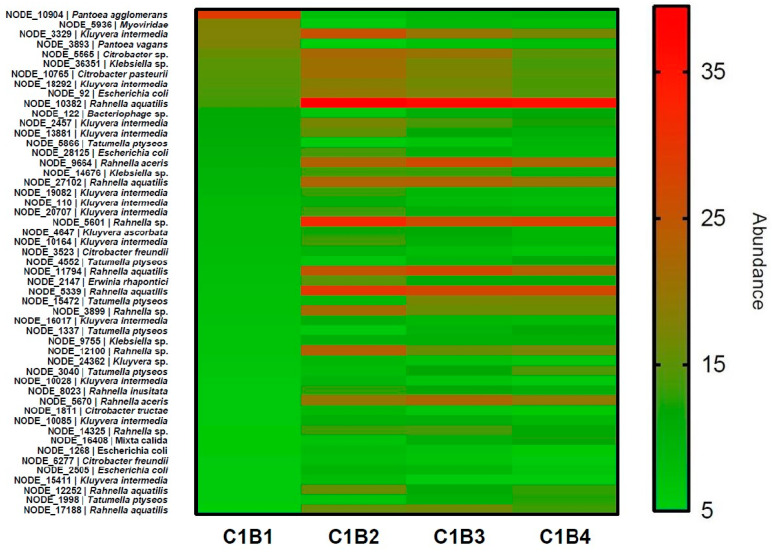
Heatmap showing the dynamics of the most abundant prophages retrieved during the first month of the fermentation process for producer B. C1 indicates the tank ID, and B1, B2, B3 and B4 are the sampling points during the fermentation process, in reference to Table 1. NODE referred to the identified prophage and the mentioned bacterial identifications represent the best host species of the prophage predicted after blast (E-value < 10^−16^; % of identity >85%). RPKM value above 5.0 was considered for the construction of the heatmap. Green to red: lowest to highest abundance.

A heatmap representation was used to visualise the dynamics of the temperate phage contigs inside bacterial metagenomes during the first month of the fermentation process for producer B (Figure 1). No heatmap could be made for producer P because the abundance was too small (RPKM max = 11.4 and 1593 values under an RPKM of 5.0 almost all equal to 0). Overall, the abundance of prophages in producer B samples tended to increase for some hosts, while other prophages fluctuated or decreased. For example, the occurrence of contig NODE_10382, affiliated to the *Rahnella aqualitis* species, increased overtime (RPKM of 13.6, 39.5, 36.4 and 35.6 in C1B1, C1B2, C1B3 and C1B4, respectively) (Table S3). Contig NODE_10904, affiliated to *Pantoea agglomerans*, decreased after C1B1 (RPKM moving from 29.2 to 7.7, 9.0 and 9.7 in C1B1, C1B2, C1B3 and C1B4, respectively) and contig NODE_3329, associated to *Kluyvera intermedia*, varied over time (RPKM passing from 17.9 to 25.9, 20.1 and 17.1 in C1B1, C1B2, C1B3 and C1B4, respectively) (Table S3). As expected, these observations of prophage occurrence altogether followed the same patterns as the levels of abundance of the bacterial genera to which they were associated. For example, the genus *Rahnella* increased overtime (total abundance of 4%, 14%, 12% and 11% in C1B1, C1B2, C1B3 and C1B4, respectively), the genus *Pantoea* decreased overtime (total abundance of 15%, 4%, 5% and 5% in C1B1, C1B2, C1B3 and C1B4, respectively), and the genus *Kluyvera* varied overtime (total abundance of 7%, 11%, 10% and 8% in C1B1, C1B2, C1B3 and C1B4, respectively) (data not shown).

Furthermore, gene identification in all the phage contigs was carried out using PROKKA. It did not reveal any gene of potential interest, as more than 95% of the coding DNA sequences were identified as hypothetical proteins (data not shown).

**Figure 2:**
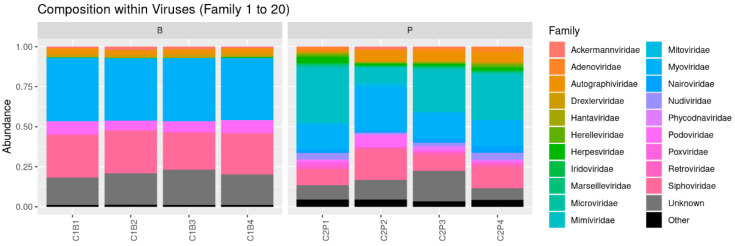
Virus taxonomic assignations at the family level during cider fermentation process for two producers (B: industrial and P: hand-crafted). C1 and C2 indicate the tank ID, and B1, B2, B3, B4, P1, P2, P3 and P4 are the sampling points during the fermentation process, in reference to Table 1.

In addition to eukaryotic and bacterial sequences, which included prophages, a marginal part of viral DNA was also recovered (only 0.08% of the total reads), indicating traces of viral particles in the tanks (Figure 2). Among this small part of the total sequences, 75% of the viral sequences in tank B belonged to phages of the *Caudoviricetes* class (former *Caudovirales* order) [33], 39% to the myophages (former *Myoviridae* family), 23 to 26% to the siphophages (former *Siphoviridae* family) and 6 to 8% to the podophages (former *Podoviridae* family) at all the time points (Figure 2). In tank P, phages of the *Caudoviricetes* class were also recovered (16 to 28% of myophages, 10 to 19% of siphophages and 1 to 7% of podophages) (Figure 2). In this tank, the most abundant recovered sequences were those of viruses belonging to the *Mimiviridae* family, infecting amoeba and protists (10 to 35%) (Figure 2).

## 4. Discussion

The interest in phageomes, has been growing in recent years [8]. However, only three studies have been strictly dedicated to the phageome of fermented foods to date [9,10,11], and one to the airborne virome in cheese-making factories [12]. No published study about the phageomes in fermented beverages is available so far. It has been shown that the phageome plays an essential role in the microbial structure and balance of ecosystems, this being especially described in the human gut microbiota [24]. In this context, it was interesting to investigate the phageome of a fermented beverage, cider, in order to assess the diversity and occurrence of free lytic phages in different cider samples throughout a fermentation process in an industrial producer (B) and in a hand-crafted producer (P). It appeared that extracting free phage DNA from cider is not easily achievable when adapting protocols used for other types of samples, such as faeces [2] and cheese [11]. Indeed, in these matrices, the level of phages is usually high (>10^9^ particles per gram), and phage DNA extraction seems thus facilitated. When using these methods in cider samples spiked with exogenous phage UCMA 21115, no phage DNA was recovered when the phage levels were under 10^7^ PFU/mL. To improve phage DNA extraction in cider, PVPP at 1% and 2% was used to limit the potential negative impact of polyphenols in cider, known to be present at high concentration levels [22]. This molecule was used in buffers for plant DNA extraction, also known for their high contents of phenolic compounds, and its use showed an improvement in DNA extraction [25]. No improvement in phage DNA extraction was achieved using PVPP in cider. Polyphenols can be a way to explain the low quantity of phages in cider due to their antiviral properties [34]. They can act either by affecting the host killing the activity of phages, such as for two phages (OE33PA and Vinitor162) infecting *Oenococcus oeni* in wine, either by interacting with the phage tail or competing with the host site of recognition [35].

Tangential flow filtration [36] and Fe-based flocculation [24], two classical methods used for the concentration of viruses from marine waters, were then tested to increase the likelihood of getting higher quantities of phages and thus of extracting phage DNA from larger volumes of cider samples. Even so, free phage DNA extraction could not be improved in cider. Considering these results, it appears that cider is a fermented matrix with probable low levels of free lytic phages, that are difficult to concentrate for extracting phageome DNA. This was corroborated in the current study as no lytic phage was isolated against 160 strains by a double layer agar plate, even after TFF and flocculation. It is probably the case for other fermented beverages, as very few lytic phages from fermented beverages are described in the literature to date. Two lytic phages targeting *O. oeni* (OE33PA and Vinitor162) and one lytic phage targeting *Gluconobacter cerinus* (GC1) have been recently described in wine [18,37,38]. Moreover, in a previous work, 120 samples (cider, apple must, crushed apples) were screened for phages targeting 160 potential strains from genera found in cider, and only one lytic siphophage UCMA 21115 was isolated [23]. This result strengthens the hypothesis that cider is a poor reservoir of free lytic phages.

Cider, due to its harsh environment, presents a less complex bacterial diversity than other fermented foods or habitats [22]. The low microbial diversity could favour the temperate lifestyle of phages (lysogeny of prophages) because it results in an absence of predator/prey dynamics [15]. As the phageome of cider seemed scarce, a particular attention was thus paid to the prophages in cider throughout the fermentation process of producer (B) and producer (P). To date, no study has been focused on the prophages in their entirety in fermented foods and beverages, even if this topic is becoming an emerging field of investigations. A recent study on wine samples from three wineries investigated the prevalence of prophages in *O. oeni* strains through the fermentation process [39]. The majority of studies investigated the distribution of prophages in bacterial genomes, as is the case for the *Lactobacillaceae* strains, members of the former *Lactobacillus* genus [40], and for the *O. oeni* strains [41]. These studies highlight that prophage diversity varies from one species to another, and that prophages are retrieved in many strains. Prophages are also recognised as markers of diversity within a species in the case of *O. oeni*, and as sources of horizontal gene transfer in *Lactobacillus*. Prophages are also known as bacterial fitness enhancers. For example, *Streptococcus pyogenes* prophage, carrying genes encoding exotoxins, increased colonisation during scarlet fever [42], and an *Escherichia coli* strain carrying a prophage with a gene encoding antibiotic resistance was more adapted to its environment under antibiotic pressure [43]. These examples demonstrate the importance of prophages on their host and the necessity to evaluate their impact and diversity in fermented foods and beverages, which is little documented to date at the scale of the whole bacterial community.

In the current study, a metagenomic approach was carried out to observe prophage signatures in bacterial metagenomes throughout the cider fermentation process. A total of 1174 putative prophage contigs were identified in silico in the bacterial metagenomes using VIBRANT software, such as has been done in cheese [44] and kefir [45]. Among them, two complete prophages were found, NODE_55 and NODE_129, matching with phages isolated from human gut metagenomic data. The majority of the prophages identified in the current study were related to bacterial genera classically associated with the apple microbiota, such as, for example, *Citrobacter*, *Klebsiella*, *Pantoa*, *Pseudomonas* and *Xanthomonas* [46,47]. The prophage dynamics in cider were correlated to their bacterial host dynamics over time. For example, some prophages, such as NODE_10382, increased, others, such as NODE_10904, decreased and some others, such as NODE_3329, fluctuated during the fermentation process. These observations were correlated to the level of bacteria of the same genera with which they were associated at each fermentation time point. The increase in the frequency of prophages over time could tend to show that lysogeny had probably been established and was predominant. Conversely, a decrease in prophage frequency could be associated to a decrease in lysogeny. Indeed, this decrease could be due to the fact that the prophages have entered a lytic cycle, and consequently, are less present in their hosts [48,49,50]. In the current study, it was not possible to determine if these prophages were active or not with this method of analysis and with the encountered difficulties to extract free lytic viral particles, as described above. In a study dealing with the virome of pigs, active and inactive prophages were distinguished with bioinformatic tools after the extraction of lytic viral particles [51]. Another possible method could be to induce prophages with mitomycine C as it has been performed on gut microbiota to study active prophages [52], bearing in mind a bias due to the fact that not all prophages are inducible. The main barrier for these types of approaches in cider, and probably in other fermented beverages, is the problematic extraction of phage DNA, due to their very low occurrence, and also to the particular physicochemical conditions encountered in these fermented beverages. Positive and negative correlations could be made in future experiments between lytic phages, prophages and bacterial proportions in cider throughout the fermentation process by combining the analyses of metabolites and food properties, as it has been described in cheese, for example [44]. For a better view of these dynamics in cider, and especially with LAB, it would be interesting to extend the timespan of the study looking at longer fermentation processes, such as cider intended to be used for the making of spirits and vinegars [53].

## 5. Conclusions

This study revealed the low occurrence of free lytic phages, and thus of phageome, in cider. This fermented beverage appears to be a poor reservoir of lytic phages in contrast to other food ecosystems, such as cheeses [11]. The presence of phages in cider is more likely to occur essentially as prophages throughout the fermentation process. Besides the fact that cider does not contain many phages, a number of questions are raised about fermented beverages. What is special about this type of ecosystem compared to other hostile environments where phages are found? Why have lytic phages not been able, at first sight, to adapt to this environment, even though it is quite rich in bacteria? Are polyphenols implicated in this situation? All these questions remain unanswered for the time being, and show the importance of looking at them in more details in order to better understand the place of phages in these ecosystems.

## Figures and Tables

**Table 1:** Cider samples collected for the study of the phageome and for the prophage investigation.

Sample ID	Producer ID (French Department)	Fermentation Time (Days)	Tank ID—Tank Volume (hL)	Sugar Concentration kg/m^3^	pH
C1B1	B (Calvados)	0	C1—120	1060	4.02
C1B2	B (Calvados)	7	C1—120	1056	4.05
C1B3	B (Calvados)	14	C1—120	1048	4.03
C1B4	B (Calvados)	27	C1—120	1036	4.01
C2P1	P (Manche)	0	C2—10	1056	3.32
C2P2	P (Manche)	12	C2—10	1049	3.35
C2P3	P (Manche)	25	C2—10	1040	3.52
C2P4	P (Manche)	36	C2—10	1036	3.58

**Table 2:** Overview of the conditions tested for the collection of the phageome from cider. Roman numerals from I to IX indicate methodological steps referred to in the text.

	Tested Conditions to Collect Viral Particles from Cider								
	I	II	III	IV	V	VI	VII	VIII Tangential Flow Filtration	IX Flocculation with FeCl_3_
Tested volumes of cider (mL)	50			20, 50, 1000				400	
Sample spiked with phage UCMA 21115 (PFU/mL)	Yes (10^4^–10^9^)							Yes (10^5^)	Yes (10^4^–10^8^)
pH adjusted to 5.5			Yes				Yes		
Precipitation with 10% PEG-8000 and 0.5 M NaCl		Yes	Yes		Yes	Yes	Yes		
Centrifugation step	Yes	Yes	Yes	Yes	Yes	Yes	Yes		
DNA extraction from	Pellet	Supernatant	Pellet	Pellet	Supernatant	Pellet	Pellet	Retentate	Flocculate

**Table 3:** Efficiency of iron-based flocculation to recover phage UCMA 21115 from cider samples.

Inoculated Quantity of Phage UCMA 21115 in 400 mL of Cider (i.e., Concentration)	Retrieved Quantity of Phage UCMA 21115 after Flocculation in Cider
4 × 10^10^ (1 × 10^8^ PFU/mL)	8.5 × 10^8^
4 × 10^9^ (1 × 10^7^ PFU/mL)	9.9 × 10^7^
4 × 10^8^ (1 × 10^6^ PFU/mL)	6.6 × 10^6^
4 × 10^7^ (1 × 10^5^ PFU/mL)	7.6 × 10^5^
4 × 10^6^ (1 × 10^4^ PFU/mL)	5.0 × 10^3^

## Data Availability

Publicly available datasets were analysed in this study. This data can be found in the NCBI SRA repository, reference number PRJNA803977.

## Supplementary Materials

The following supporting information can be downloaded at: https://www.mdpi.com/article/10.3390/microorganisms10061203/s1, Figure S1: Agarose gels of phage DNA extraction from cider adjusted to pH 5.5 spiked with phage UCMA 21115. Figure S2: Microbial taxonomic assignations at the domain level during the cider fermentation process for two producers (B: industrial and P: hand-crafted). Figure S3: Eukaryota taxonomic assignations at the genus level during the cider fermentation process for two producers (B: industrial and P: hand-crafted). Figure S4: Bacterial taxonomic assignations at the family level during cider fermentation process for two producers (B: industrial and P: hand-crafted). Table S1: Percentage of the number of reads attributed to Super-kingdoms in NCBI. Table S2: Bacterial strains used to isolate bacteriophages. Table S3: Occurrence of prophages for producer B and P.
