# Supplementary figures and images for: Investigation of the Phageome and Prophages in French Cider, a Fermented Beverage
Source: Microorganisms. 2022 Jun 12;10(6):1203. doi: 10.3390/microorganisms10061203 (PMC9230842; doi:10.3390/microorganisms10061203)

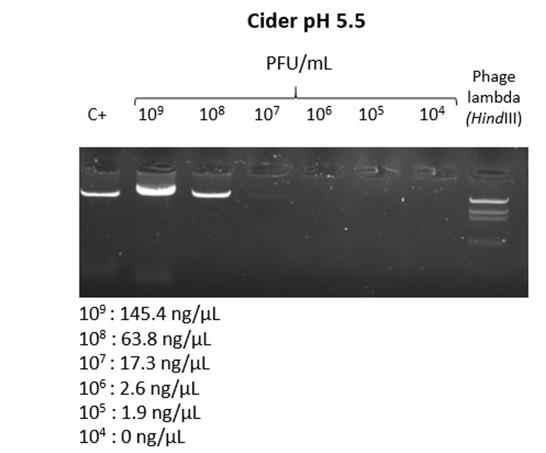

Supplement: Supplementary file 1 [file microorganisms-10-01203-s001.zip › Figure S1.jpg]

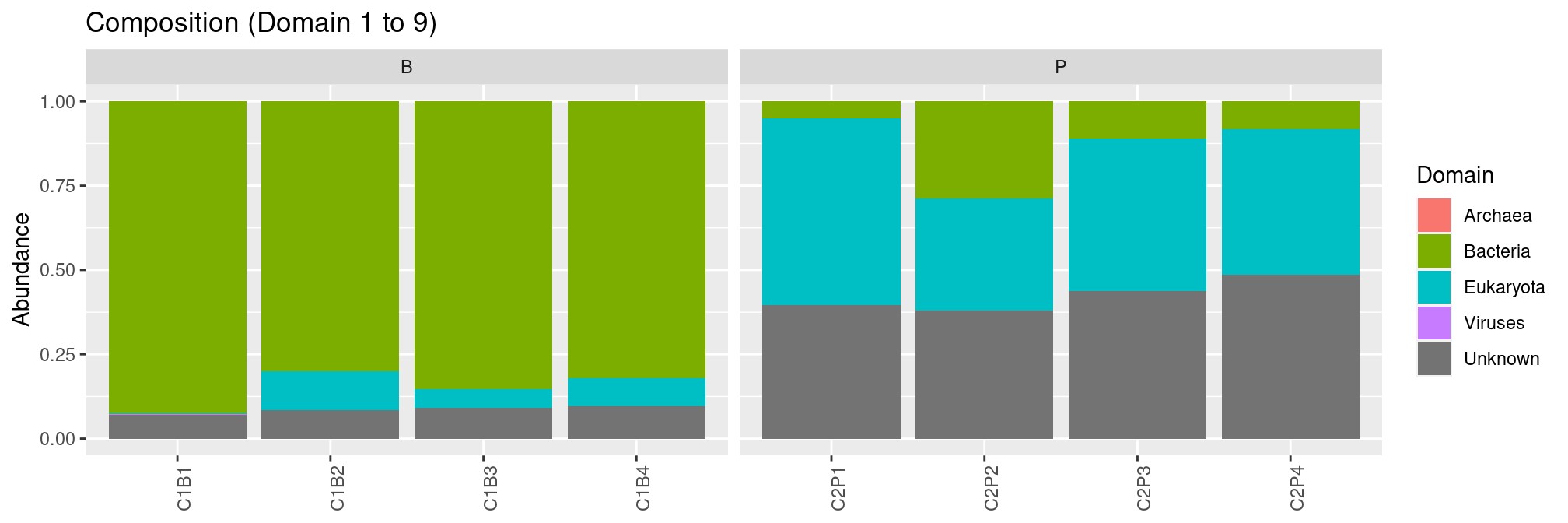

Supplement: Supplementary file 1 [file microorganisms-10-01203-s001.zip › Figure S2.jpg]

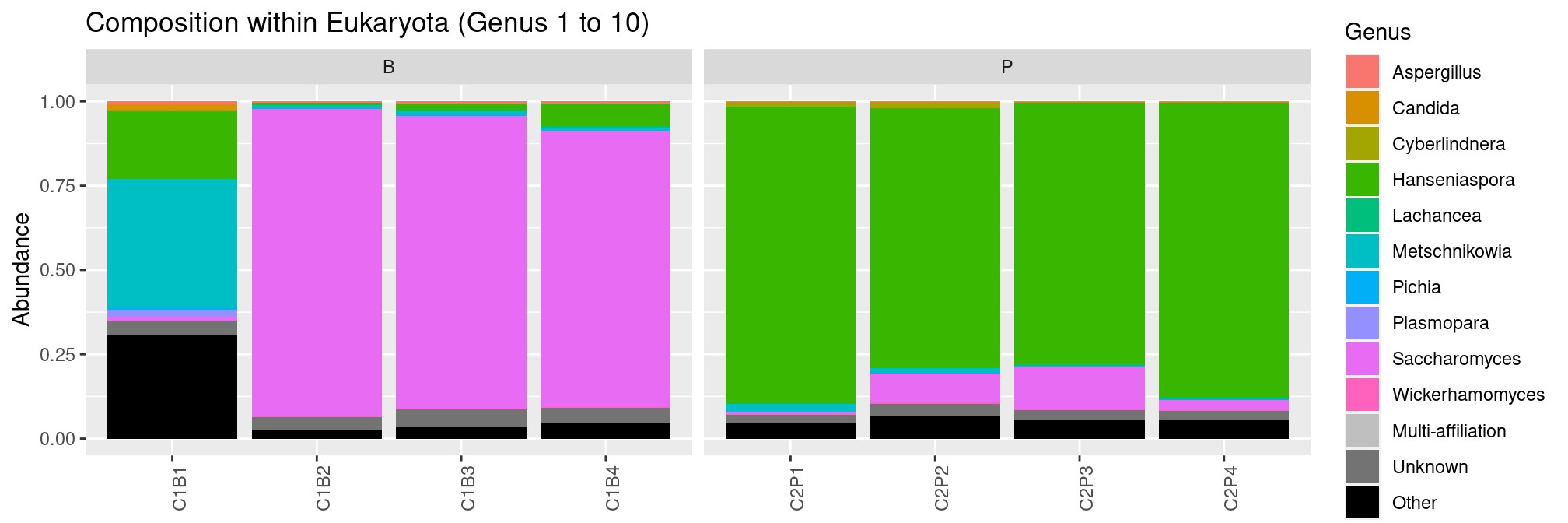

Supplement: Supplementary file 1 [file microorganisms-10-01203-s001.zip › Figure S3.jpg]

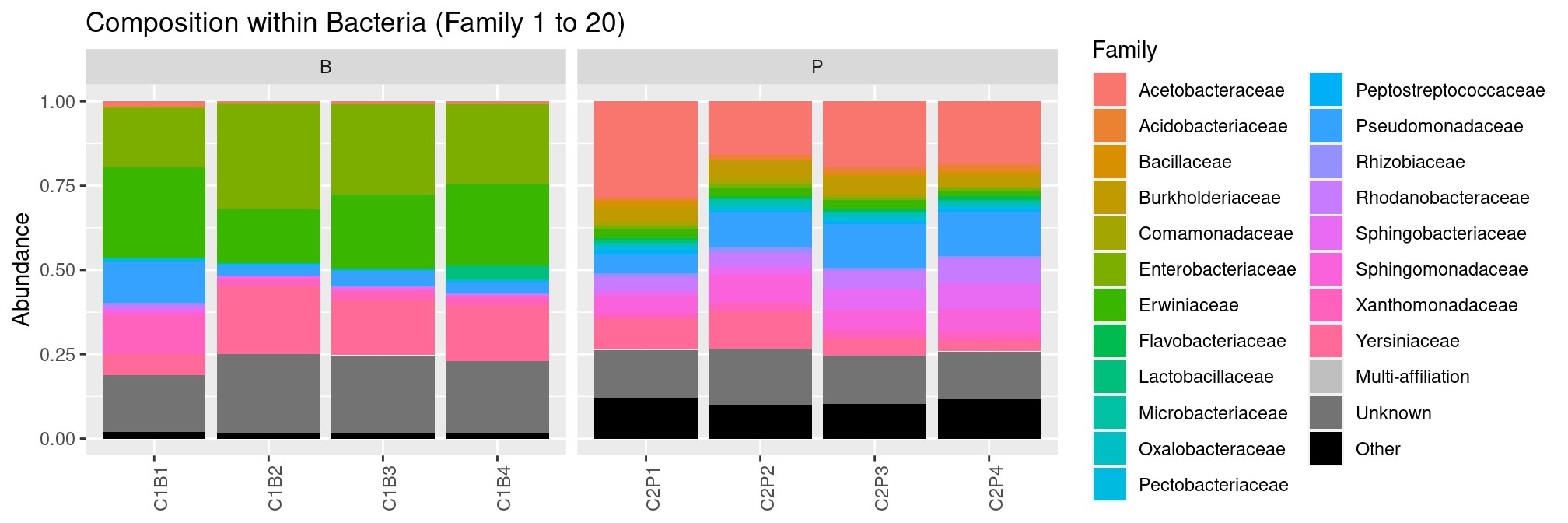

Supplement: Supplementary file 1 [file microorganisms-10-01203-s001.zip › Figure S4.jpg]
